# Acute mitral regurgitation after transcatheter tricuspid valve replacement – case report

**DOI:** 10.1093/ehjcr/ytag391

**Published:** 2026-06-02

**Authors:** Barbara Rubinic, Carolina Göttsche Esperanca Clara, Maria Ivannikova, Tanja K Rudolph, Johannes Kirchner

**Affiliations:** Department of General and Interventional Cardiology/Angiology, Ruhr-Universität Bochum, Herz- und Diabeteszentrum NRW, Georgstraße 11, Bad Oeynhausen 32545, Germany; Department of General and Interventional Cardiology/Angiology, Ruhr-Universität Bochum, Herz- und Diabeteszentrum NRW, Georgstraße 11, Bad Oeynhausen 32545, Germany; Department of General and Interventional Cardiology/Angiology, Ruhr-Universität Bochum, Herz- und Diabeteszentrum NRW, Georgstraße 11, Bad Oeynhausen 32545, Germany; Department of General and Interventional Cardiology/Angiology, Ruhr-Universität Bochum, Herz- und Diabeteszentrum NRW, Georgstraße 11, Bad Oeynhausen 32545, Germany; Department of General and Interventional Cardiology/Angiology, Ruhr-Universität Bochum, Herz- und Diabeteszentrum NRW, Georgstraße 11, Bad Oeynhausen 32545, Germany

**Keywords:** Case Report, Diastolic Dysfunction, Echocardiography, Mitral Regurgitation, Transcatheter Tricuspid Valve Replacement

## Abstract

**Background:**

Transcatheter tricuspid valve replacement (TTVR) has emerged as a therapeutic option for patients with severe tricuspid regurgitation (TR) who are ineligible for surgery.

**Case Summary:**

We report the first case of acute development and transient resolution of severe mitral regurgitation (MR) following TTVR device implantation. Baseline transthoracic echocardiography (TTE) showed torrential TR and mild to moderate MR. Immediately after TTVR-device implantation, transoesophageal echocardiography (TOE) revealed severe MR. Over the following days, MR gradually improved, and TTE at discharge showed only mild to moderate MR.

**Discussion:**

This case illustrates that abrupt elimination of TR can lead to acute worsening of MR, likely precipitated by an abrupt increase in left ventricular preload in the presence of diastolic dysfunction. In conclusion, caution is warranted in patients with pre-existing left ventricular dysfunction undergoing TTVR, as the procedure may exacerbate functional MR. Periprocedural volume unloading may help ameliorate the impact of acute haemodynamic changes.

Learning points

- TTVR devices should be used with caution in patients with impaired LV function and concimmattent valvular disease.
- The mitral valve should be carefully assessed prior to and after the TTVR procedure.

## Introduction

Transcatheter tricuspid valve replacement (TTVR) has emerged as a therapy option for patients with symptomatic severe tricuspid regurgitation (TR) who are ineligible for cardiac surgery.^1^ In contrast to transcatheter edge-to-edge repair (T-TEER), TTVR leads to abrupt and often nearly complete elimination of TR. As a consequence, rigorous haemodynamic shifts are imposed on the right, and consequently, on the left ventricle.^2^ Here we present a patient who showed acute and transiently reversible mitral regurgitation after TTVR-device implantation.

## Summary figure

**Figure:**
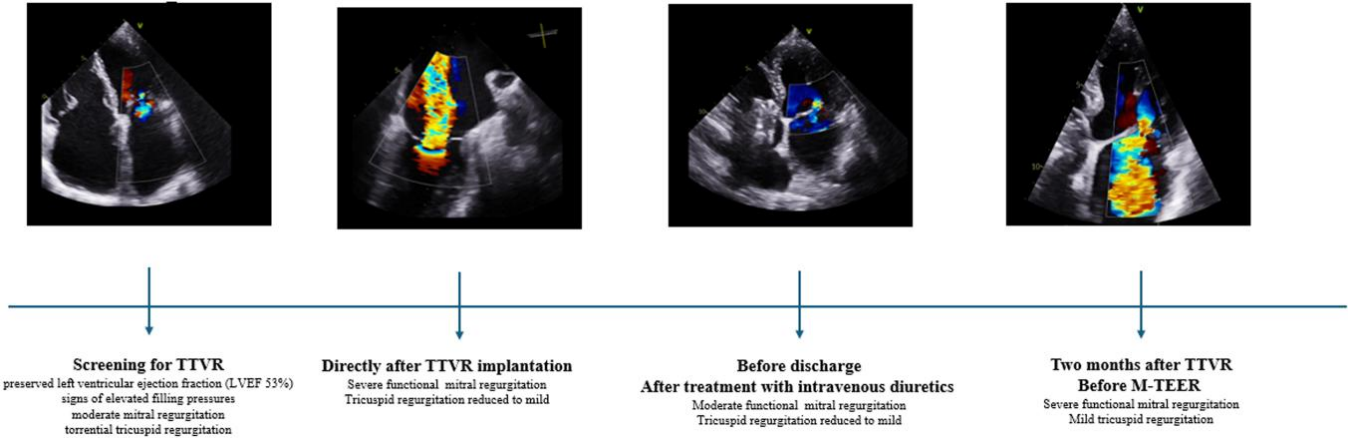


## Case presentation

An 87-year-old woman presented with dyspnoea on exertion classified as New York Heart Association (NYHA) functional class III. She had already been hospitalized multiple times due to heart failure with preserved ejection fraction. Relevant comorbidities included persistent atrial fibrillation, hypertension, dyslipidaemia, and chronic kidney disease class G3b according to Kidney Disease: Improving Global Outcomes classification (KDIGO). Further laboratory tests showed an increased *n*-terminal pro-brain natriuretic peptide (NT-proBNP) of 2050 pg/mL, and normal bilirubin levels (0.76 mg/dL). The electrocardiogram (ECG) showed atrial fibrillation, with an average of 75 bpm.

**Figure 1:**
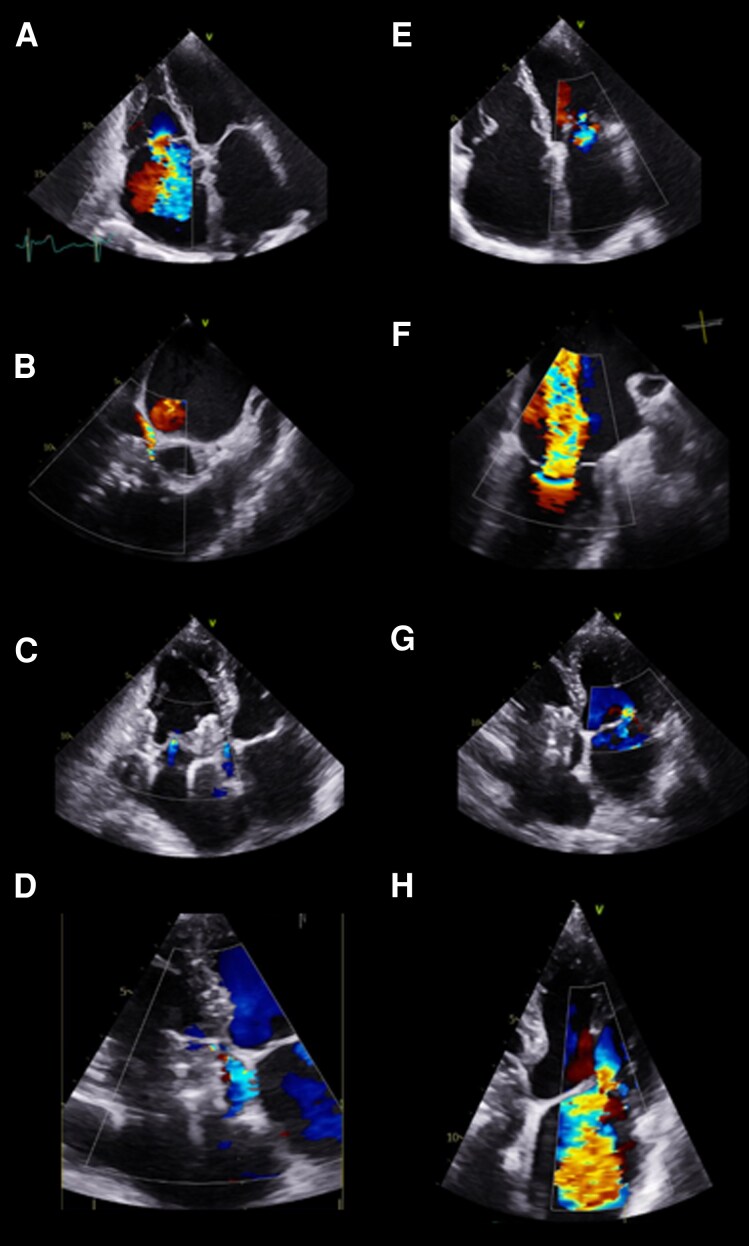
Transthoracic echocardiogram and transoesophageal echocardiography showing tricuspid regurgitation (a – TTE at baseline, B – TOE after TTVR implantation, C – TTE at discharge, D – TTE two months after TTVR) and mitral regurgitation (E – TTE at baseline, F – TOE after TTVR implantation, G – TTE at discharge, H – TTE two months after TTVR).

The baseline transthoracic echocardiogram (TTE) revealed a preserved left ventricular ejection fraction (LVEF 53%) with signs of diastolic dysfunction. Moderate mitral regurgitation (MR) was present, with an effective regurgitant orifice area (EROA) of 0.2 cm^2^ and a calculated regurgitant volume of 30 mL, along with mild aortic regurgitation. Right ventricular function was reduced (Tricuspid annular plane systolic excursion [TAPSE] 16 mm, S´ 7 cm/s, 3D right ventricle ejection fraction 34%). Additionally, secondary atrial tricuspid regurgitation (TR) was graded as torrential (EROA 1.4 cm^2^, RV 79 mL, coaptation gap 6 mm, *Figure 1*), and was assumed to be the most likely driver to the patient’s symptoms and previous episodes of cardiac decompensation.

Surgical risk was elevated, as expressed by a TRI-Score^3^ of 6, corresponding to an estimated in-hospital mortality risk of 22% for isolated tricuspid surgery. Furthermore, the TRIVALVE Score^4^ of 2.5 also presented an elevated risk after transcatheter tricuspid valve intervention. Therefore, the interdisciplinary heart team recommended transcatheter tricuspid valve intervention for symptom relief. Due to the unfavourable anatomy and large coaptation gap, the patient was not eligible for transcatheter edge-to-edge repair. Consequently, following confirmation of anatomical suitability by full-cycle cardiac computed tomography, a TTVR device implantation was planned.

Before TTVR, the patient was treated with intravenous diuretics for optimal prehabilitation. The procedure was performed under general anaesthesia and guided by transoesophageal echocardiography (TOE) and fluoroscopy. Device implantation was uneventful, and TR was reduced from ‘torrential’ to ‘mild’, caused by a small paravalvular leak (*Figure 1*.). Directly after TTVR implantation, periprocedural TOE revealed severe functional MR. The MR was caused by a central jet with (EROA 0.8 cm^2^ and RV of 38 mL), and a posterior jet (EROA of 0.4 cm^2^, RV 25 mL, *Figure 1*). These findings were accompanied by a visible coaptation gap in the A2/P2 segment of the mitral valve, corresponding to severe MR.

After the procedure, the patient was monitored in the intensive care unit (ICU). Due to mild hypotension, a minimal dose of vasopressor (maximum norepinephrine dosage 0.032 µg/kg/min) was administered but was gradually tapered off over the following hours when haemodynamic stability was achieved. The patient was extubated three hours post-procedure, requiring only 2–4 L/min of oxygen via nasal cannula. There were no signs of pulmonary fluid congestion. Laboratory findings showed a peak NT-proBNP of 5090 pg/mL.

On admission to the ICU and the following day, echocardiography revealed ameliorated but still severe mitral regurgitation. After 48 h, the patient was transferred to the normal ward and gradually mobilized over the subsequent days. The patient was treated with intravenous diuretics, achieving net weight loss of approximately 1.4 kg during the hospital stay. At discharge, 6 days after device implantation, TTE showed the TTVR-device *in situ* with minimal paravalvular regurgitation. Moreover, only mild mitral regurgitation (EROA 0,2 cm^2^, RV 25 mL) and mild aortic regurgitation were present.

Three months after TTVR, the patient experienced progressive dyspnoea and was hospitalized due to congestive heart failure. Echocardiography showed a severe secondary mitral regurgitation (*Figure 1*.). Four months after TTVR, the severe MR was successfully treated with transcatheter mitral valve edge-to-edge therapy, resulting in only mild residual MR.

## Discussion

Here, we report the first case of acute development and transient resolution of severe MR following TTVR. Despite the sudden emergence of acute MR, the patient was haemodynamically and respiratory stable.

We hypothesize that the sudden obliteration of tricuspid regurgitation (TR)—from ‘torrential’ to ‘mild’—led to an acute increase in RV forward flow. This abrupt volume shift and rapid increase in pulmonary circulation subsequently raised left ventricular (LV) preload. We hypothesize that this acute increase in LV preload, combined with the underlying diastolic dysfunction, led to the worsening of pre-existing, but moderate, functional MR. Similarly, the TriValve registry observed that 11% of patients undergoing tricuspid valve intervention have worsening of mitral regurgitation at follow-up.^5^

Furthermore, our patient hat mildly impaired kidney function. The absence of severely impaired kidney function and acute kidney failure probably mitigated the resolution of the volume overload.

Patients undergoing TTVR are typically advanced in age, have multiple comorbidities,^6^ and many present with multivalvular disease.^7^ Secondary MR may be underestimated in the presence of severe TR due to reduced preload.^8^ Although there are lower EROA thresholds applied in secondary MR to assess for severe regurgitation^7^ and our patient had still only a moderate MR in preprocedural echocardiography, one might consider treating the MR earlier. TTVR device implantation might unmask underlying LV dysfunction due to an abrupt increase in LV preload. In patients with reduced LV function, this volume shift may jeopardize haemodynamic stability and potentially lead to cardiogenic shock. Therefore, TTVR devices should be used with caution in patients with compromised right ventricular (RV) function, as well as those with dysfunctional LV and concomitant valvular disease. Optimization of volume status with diuretics has been shown to reduce the coaptation gap in patients undergoing T-TEER,^9^ but it is yet to be investigated how it correlates with the procedural success after TTVR. Furthermore, volume unloading before the procedure may help alleviate the occurrence of sudden MR exacerbation. Nevertheless, caution is warranted during follow-up, as increased LV preload after TTVR may predispose to recurrent hospitalization.

## Conclusion

We present a case of a patient undergoing TTVR with acute and transiently reversible periprocedural worsening of mitral regurgitation. The increased RV forward flow most likely caused a substantial rise in LV preload leading to an acute worsening of MR in the setting of underlying diastolic dysfunction. Although severe MR improved in the days following TTVR, these patients remain at high risk for rehospitalization due to recurrent MR–related heart failure.

## Data Availability

All data are incorporated into the article.

## Lead author biography

I earned my medical degree from the University of Zagreb School of Medicine. Furthermore I advanced my academic qualifications by completing a Master of Science in Chemistry at the Faculty of Science, University of Zagreb.I am currently pursuing specialist residency training in Cardiology at the Heart and Diabetes Center NRW, Germany.
